# Comparison of Postoperative Pain and Analgesic Requirements Between Laparoscopic and Open Hernia Repair in Children

**DOI:** 10.1007/s00268-021-06295-x

**Published:** 2021-08-30

**Authors:** Eilidh S. Bruce, Sesi A. Hotonu, Merrill McHoney

**Affiliations:** 1 University of Edinburgh, Edinburgh, UK; 2 Department of Paediatric Surgery, Royal Hospital for Sick Children, Sciennes Road, Edinburgh, EH9 1LF UK

## Abstract

**Background:**

This study analyses the impact of anaesthetic blockade and intraperitoneal local anaesthetic infiltration on paediatric laparoscopic inguinal hernia repair.

**Method:**

A retrospective review of paediatric laparoscopic hernia repairs versus open repairs. Anaesthetic blockade, analgesic consumption and postoperative pain scores were compared between groups.

**Results:**

155 children underwent laparoscopic repair, 150 underwent open repairs. Median age was 7.2 months (16 days–14 years) in the laparoscopic group, 6 months (17 days–13 years) in the open group. Anaesthetic blockade varied significantly; 62.7% of open cases had caudal blockade compared to 21.6% laparoscopic (*p* < 0.001). A subset of laparoscopic patients had peritoneal local anaesthetic infiltration. 10.1% of laparoscopic cases required recovery analgesia, compared to 1.3% of open cases (*p* = 0.001). Postoperative analgesic consumption was significantly higher in the laparoscopic group. Peritoneal infiltration reduced analgesic consumption in the laparoscopic group (*p* = 0.038). Age < 2 was associated with use of caudal (*p* < 0.001), which reduced analgesic consumption.

**Conclusions:**

Laparoscopy was associated with increased use of recovery analgesia. Caudal reduced the need for rescue and postoperative analgesia. Intraperitoneal infiltration of local anaesthetic is associated with reduced postoperative analgesia in laparoscopy. In suitable patients undergoing laparoscopic surgery, combination caudal and peritoneal infiltration may prove a useful adjunctive analgesic strategy.

## Introduction

Minimally invasive surgery (MIS) is becoming increasingly utilised in paediatric practise, and has been proven to be safe and effective [1, 2]. Initial reports revoked the often cited benefits of laparoscopy on cosmesis, pain scores and length of stay in the treatment of children [3, 4]. Laparoscopy has the specific benefit in inguinal hernias for the detection and repair of synchronous defects in the context of bilateral pathology, and in the assessment of other differential diagnoses (for example examination of female internal genitalia) [5].

Currently, the literature surrounding paediatric laparoscopic inguinal hernia repair focuses primarily on comparing surgical outcomes [6]. Little published research on pain following laparoscopic surgery exists. Pain following laparoscopic surgery can originate from multiple sources including incisional pain and shoulder tip pain from diaphragmatic irritation secondary to CO_2_ insufflation [7]. Open surgery usually involves a single incision that is often larger than that needed for the laparoscopic approach and involves more tissue dissection [5].

There is a lack of evidence on pain encountered post paediatric laparoscopic hernia repair compared to its open counterpart, or indeed on the optimum analgesic strategies that should be employed in laparoscopic repair. Our primary aim was thus to compare open and laparoscopic inguinal hernia repair in terms of postoperative pain outcomes and analgesic requirements. Secondly, we aimed to evaluate the effect of intraperitoneal local anaesthetic infiltration on these outcomes.

## Materials and methods

### Inclusion criteria

A retrospective audit of all children undergoing laparoscopic inguinal hernia repair over a 5-year period versus a consecutive and contemporaneous cohort of open inguinal hernia repairs over two years. The audit was registered with our local quality improvement team and approved by the University of Edinburgh ethics committee.

Data were retrieved from patient clinical and operative notes (held electronically in a local database), and paper nursing and anaesthetic charts. The type of operation performed was dependent solely on individual surgeons’ preferences. The laparoscopic and open procedures were performed using standard approaches previously described in a clinical outcome paper [6]. For clarity of data analysis, we excluded all redo hernia repairs, and all conversions from one modality to another (laparoscopic to open, and vice versa).

### Data collection and outcome measures

The following patient demographics were collected: age at operation, gender, birth gestation, weight, hernia site and clinical presentation (elective, emergency). Intraoperative variables recorded were the type of anaesthetic block, use of opiate and non-opiate analgesics and anaesthetic technique. Outcome measures were need for analgesia in recovery, analgesic use and pain scores.

### Assessment of pain and analgesic consumption

The “Analgesic Consumption Score” (Table 1) was used to provide a comparable measure of analgesic consumption.

**Table 1:** Analgesic consumption score

Analgesia	Score
No analgesia	0
Non opioid (paracetamol, non-steroidal anti-inflammatory) ± adjuvant	1
Weak opioid ± non opioid ± adjuvant	2
Strong opioid ± non opioid ± adjuvant	3

Weak opioid refers to a medication with a mild to moderate effect on opioid receptors such as codeine. Strong opioid refers to a medication with a strong effect on opioid receptors such as morphine. Adjuvants refer to any medications which do not fall into the non-steroidal, opiate or paracetamol category such as gabapentin

Scores were derived using the “World Health Organisation (WHO) Pain Relief Ladder” [8]. Intraoperative analgesia was defined as analgesia administered exclusively during the time under anaesthetic. Postoperative analgesia was defined as administered after departure from recovery, until discharge from hospital (usually 4–24 h). Rescue analgesia was defined as the need for supplementary analgesia in the recovery room to allow return to the ward and was analysed independently.

Maximum postoperative pain score was documented using the validated FLACC (Face, Legs, Activity, Cry, Consolability) scale [9], providing a score on a scale of 0–10. We further stratified patients into two age groups (<2 years old and ≥2 years old) given differences in available analgesics and communication abilities between these age groups.

### Statistical analysis

Statistical analysis was performed using SPSS statistics for windows (IBM Corporation 2015, version 24, Armonk, NY). Results were described using percentages, mean or median and range. Data were analysed using *t*-test, Mann Whitney *U* test, Pearson’s chi-squared (*X*^2^) test and Fisher’s exact test. Logistic regression was performed to correlate outcome measures with patient demographics. Multivariate analysis with post hoc analysis was performed to assess the effect of confounding factors on outcome measures between subjects. A *p*-value of < 0.05 was considered significant.

## Results

### Patient demographics

We identified 305 patients. Of these, 155 had undergone laparoscopic inguinal hernia repairs, and 150 had an open repair. Patient demographics are summarised in Table 2.

**Table 2:** Patient demographics reported analysed by operative group (open versus laparoscopic)

	Open (*n* = 150)	Laparoscopic (*n* = 155)	*p* value	Odds ratio	95% CI
Age(median, range)	6 months (17 days–13 years)	7.2 months (16 days–14 years)	0.29	1.14	0.89–1.46
Weight (median, range)	7.6 kg (2.1–48.4)	7.8 kg (2.1–58.2)	0.29	0.96	0.89–1.04
Gender			0.26	0.69	0.37–1.30
Male	125 (83.3%)	119 (76.8%)			
Female	25 (16.7%)	36 (23.2%)			
Gestation (median, range)	38 weeks (24–42)	37 weeks (23–43)	0.52	0.98	0.92–1.05
Side of hernia			<0.001		
Right	95 (63.3%)	64 (42.4%)			
Left	44 (29.3%)	28 (18.5%)			
Bilateral	11 (7.3%)	59 (39.1%)			
Presentation			0.004	2.72	1.37–5.39
Elective	100 (66.7%)	128 (84.8%)			
Emergency	50 (33.3%)	23 (15.2%)			
Length of stay			0.49	0.87	0.57–1.31
Day case	74 (49.3%)	79 (52.3%)			
Inpatient	76 (50.7%)	72 (47.7%)			

Comparison of patient demographics between the open and the laparoscopic groups showed no significant difference in age, sex, weight at operation, or gestational age (Table 2). As expected, there was a predominance of right-sided inguinal hernias in both groups, and more bilateral operations in the laparoscopic group. There was no difference in patient outcomes between unilateral and bilateral repairs. In both the laparoscopic and open groups, the majority of hernia repairs were performed electively. The choice of operative modality utilised in both elective and emergency cases was solely based on individual surgeons’ preference and skill.

### Comparison of intraoperative analgesia and analgesic consumption by operative modality

Method of operation was significantly associated with the type of anaesthetic blockade employed (Table 3); 92.1% of open hernia repairs received some sort of anaesthetic block (caudal or regional) compared to 23.7% of laparoscopic cases (*p* < 0.001). In both groups, caudal anaesthetic was the most commonly utilized block compared with other modalities (*n* = 94 or 62.7% of open cohort and *n* = 34 or 21.6% of laparoscopic cohort, *p* < 0.001).

**Table 3:** Pain scores and analgesic consumption by operative modality

	Open (*n* = 150)	Laparoscopic (*n* = 155)	*p* value
Analgesic block			
None	8.0%	76.4%	<0.001
Ilioinguinal	28.7%	0.7%	
Caudal	62.7%	21.6%	
Transversus abdominis plane (TAP)	0.7%	1.4%	
Intraoperative analgesia consumption score (median, interquartile range)	1 (0–1)	3 (1–3)	<0.001
Rescue analgesia			
Yes	1.3%	10.1%	0.001
No	98.7%	89.9%	
Maximum postoperative pain score (median, interquartile range)	0 (0–2)	0 (0–2)	0.14
Postoperative analgesia consumption score (median, interquartile range)	1 (1–2)	2 (1–2)	0.005

Intraoperative consumption scores differed significantly between the two groups; open inguinal hernia repair was associated with a lower intraoperative consumption compared to laparoscopic repair (median 1 (interquartile range 0–1) versus median 3 (interquartile range 1–3), *p* < 0.001). Postoperative analgesia consumption scores for open cases (median 1, interquartile range 1–2) were significantly less than for laparoscopic cases (median 2, interquartile range 1–2; *p* = 0.005).

### Comparison of postoperative pain and analgesic consumption by anaesthetic block

When analysed according to anaesthetic blockade employed, in both groups use of caudal anaesthesia were associated with significantly lower postoperative pain scores, and need for rescue analgesia compared to other types of block or no block at all. 11.3% of patients with no anaesthetic block required recovery analgesia compared to 1.6% of patients with caudal anaesthesia (*p* < 0.001).

**Fig. 1:**
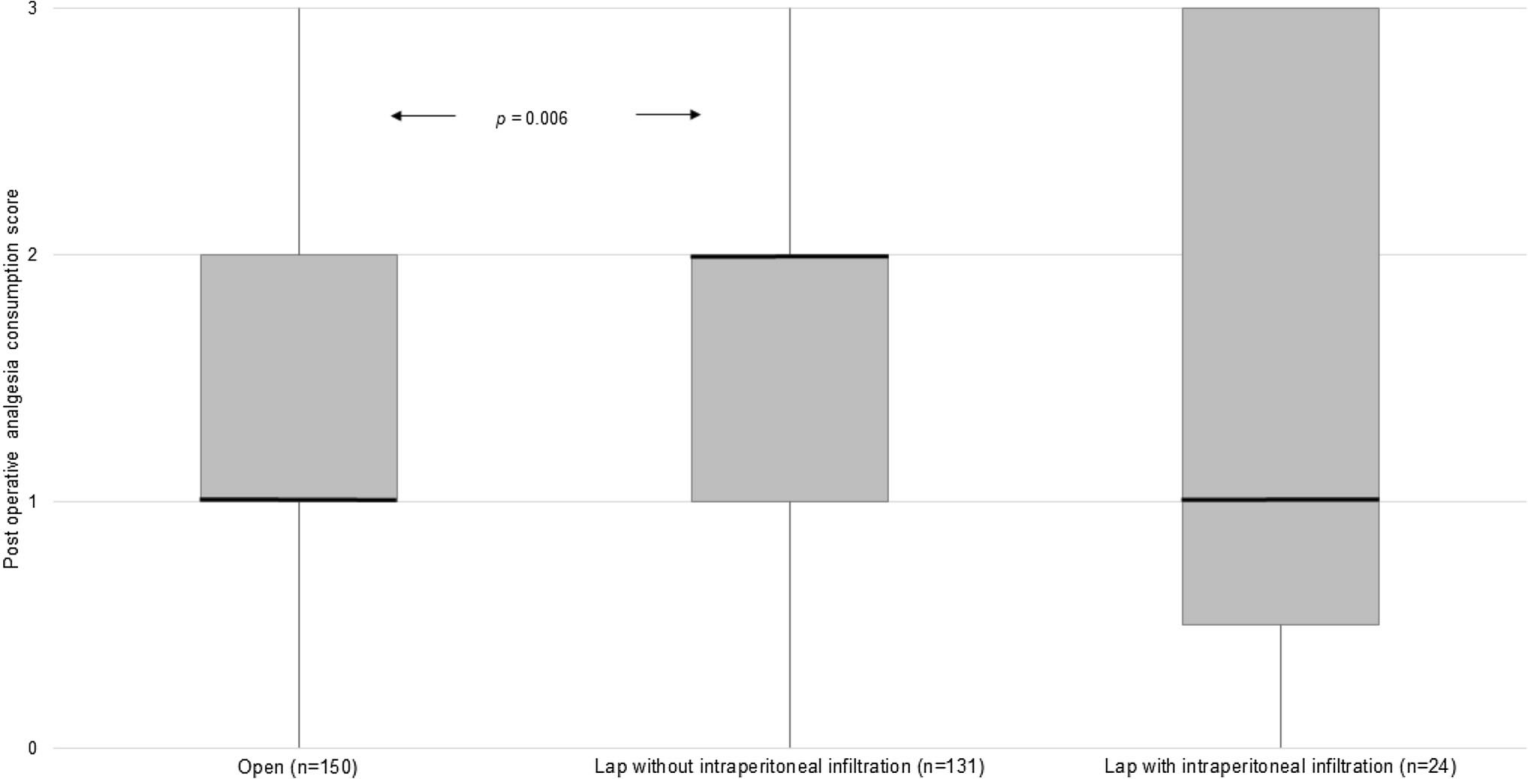
Box plot showing postoperative analgesia consumption score based on method of operation and administration of intraperitoneal local anaesthetic. Bars represent median, boxes represent interquartile range and whiskers represent range

A small number of patients in the laparoscopic group (*n* = 24) received intraperitoneal infiltration of local anaesthesia (calculated weight-based dose of bupivacaine with adrenaline). There was no difference in the need for rescue analgesia in patients who had intraperitoneal infiltration of local anaesthetic compared to those who did not (9.1% compared to 10.3%). There was however a significant difference in pain score between those laparoscopic cases, which received peritoneal infiltration and those who did not (*p* = 0.038). Post hoc pairwise comparisons revealed higher postoperative analgesic consumption scores in the laparoscopic group without peritoneal infiltration (median 2, interquartile range, 1–3) compared to the open group (median 1, interquartile range, 1–2; *p* = 0.006). Scores comparing the other groups were not significantly different (Fig. 1).

### Comparison between age groups

We analysed the data in two age groups: <2 years old and ≥2 years old (Table 4). There was no significant difference in operative method between age groups. As expected, a significantly greater proportion of the <2 year age group received caudal analgesia compared to the ≥2 year age group (*p* < 0.001). There was no significant difference in need for rescue analgesia and median postoperative pain score between the two age groups, but analgesic consumption was higher in the older patients.

**Table 4:** Comparison between age groups (<2 years old, ≥2 years old)

	<2 years old (*n* = 187)	≥2 years old (*n* = 118)	*p* value
Operative modality			
Open (*n*)	90	60	0.72
Laparoscopic (*n*)	97	58	
Analgesic block			
None	4.3%	10.2%	0.06
Regional	35.8%	77.9%	0.24
Caudal	59.9%	11.9%	< 0.001
Intraoperative analgesia score (median, interquartile range)	1 (0–1)	2 (1–3)	0.01
Rescue analgesia (percentage)	4.9%%	7.0%	0.45
Maximum postoperative pain score (median, interquartile range)	0 (0–2)	0 (0–2)	0.18
Postoperative analgesia consumption (median, interquartile range)	1 (1–2)	2 (1–2)	0.011

On analysis of the <2 year age group by anaesthetic block and operative method, open hernia repair was associated with increased use of caudal anaesthesia compared to laparoscopic repair (*n* = 81 versus *n* = 31, *p* < 0.001). The use of caudal anaesthesia in the <2 year age group was associated with significantly lower median postoperative pain score (median 1 versus median 5; *p* = 0.007) and use of rescue analgesia (*p* = 0.02). These findings were not observed in the ≥2 year old age group.

### Effect of confounding variables on outcomes

A multivariate analysis was performed using all confounding variables to determine the effects of each on the reported outcomes (Table 5). Laparoscopic surgery and lack of caudal analgesia were associated with increased need for rescue analgesia. Maximum pain score was independently related to age. Interestingly all factors were significantly correlated with the analgesic consumption score.

**Table 5:** Multivariate analysis of dependant variables associated with interpatient differences in outcome measures in the study population

Outcome measure	Dependent variable	*p* value
Rescue analgesia	Operative modality	.002
	Caudal	.012
	Age	.210
	Intraperitoneal infiltration of local anaesthesia	.589
Maximum pain score	Operative modality	.920
	Caudal	.098
	Age	<0.001
	Intraperitoneal infiltration of local anaesthesia	.948
Analgesic consumption score	Operative modality	<0.001
	Caudal	<0.001
	Age	<0.001
	Intraperitoneal infiltration of local anaesthesia	<0.001

## Discussion

Current data on the feasibility and outcomes of laparoscopic inguinal hernia repair in children exists mainly on operative parameters with little focus on pain [5, 10]. Our data suggest a relationship between modality of intraoperative analgesia utilised in paediatric laparoscopic hernia surgery and postoperative analgesic requirements. Increased use of caudal anaesthetic seems to specifically reduce the need for rescue analgesia in recovery. In the laparoscopic group, intraperitoneal infiltration of local anaesthetic seems to be associated with reduced analgesic consumption postoperatively. The data suggest an overall additive effect of analgesic modalities, which reduces overall postoperative analgesic consumption.

In 2005, Chan et al*.* observed that laparoscopic repair was associated with less pain (using a scale similar to the FLACC score used in this study) and less postoperative paracetamol consumption [10]. Similar results were elicited in a slightly older paediatric population (>6 years), in a randomised study by Celebi et al. in the management of bilateral hernias [11]. However, in a 2014 Helsinki group study of 89 patients, 79% of laparoscopic cases required postoperative analgesia compared to 42% undergoing open repair [5]. We observed a similar phenomenon in our series. It is worth noting however that heterogeneous reporting of analgesic consumption and pain outcomes makes direct comparisons between studies unreliable.

There was greater use of anaesthetic blockade in patients undergoing open hernia repair in this study; 92% of open cases had some form of anaesthetic block, compared to 23.6% of laparoscopic cases. Of note was the association between caudal anaesthesia and reduction in rescue analgesia and analgesic consumption; we observed across the two operative modalities an up to 90% reduction in the use of rescue analgesia in patients with caudal anaesthesia compared to those without. The increased use of caudal anaesthesia in the open group compared to the laparoscopic group could indeed explain some of the differences in postoperative pain and analgesic consumption observed in our series. A recent systematic review by Shanthanna et al. supports this, concluding that patients with caudal blockade require less rescue analgesic compared with other regional blockade techniques in open paediatric inguinal surgeries [12]. This is however contradicted by Baird et al. who demonstrated no difference in pain scores or rescue analgesia consumption when comparing caudal blockade with alternative analgesic strategies [13].

The second arm of this study aimed to compare analgesic consumption and pain outcomes in those laparoscopic cases receiving intraperitoneal local anaesthetic infiltration (IPLA) versus those without local anaesthetic infiltration. Our results in this comparison are promising, yet affected by the small sample size (*n* = 24). We have shown a significant difference in maximum postoperative pain score and analgesic consumption in the IPLA group compared to those laparoscopic cases without infiltration. Numerous adult studies report the benefits of intraperitoneal local anaesthetic infiltration in laparoscopic surgery; Alkhamesi et al. found significant reduction in port site and shoulder tip pain in adult laparoscopic surgery following application of intraperitoneal aerosolized bupivacaine [14]. In 2016, Hamill et al*.* identified 4 trials of IPLA in paediatric laparoscopic surgery which suggest its benefit in the reduction of pain scores, opioid use and requirement for rescue analgesia [15]. It has been suggested that the mechanism behind this phenomenon is the neutralization of the acidic carbon dioxide widely used as an insufflation agent by the relatively alkaline local anaesthetic agent, hence reducing diaphragmatic and intraperitoneal irritation [16, 17]. Indeed, this theory is supported by the successful reduction of postoperative pain from laparoscopic surgery through the use of intraperitoneal sodium bicarbonate spray in some studies [18–21]. Gupta et al. suggest a multimodal approach to anaesthesia and analgesia in laparoscopic day case procedures, with individual sources of pain (incisional, insufflation) addressed using the best technique appropriate for the patient [22].

When analysed by age, we observed increased use of caudal anaesthesia in the < 2 year age group compared to the ≥ 2-year-old age group. It is worth noting that caudal analgesia was more likely to be used in the < 2-year-old age group. There was a significant reduction in postoperative and recovery analgesic requirements in the patients receiving caudal analgesia in the < 2-year-old age group compared to those without; similar findings were observed by Tobias et al. [23]. Our observations are mirrored in several studies in the adult population where regional or spinal anaesthesia has been employed during abdominal surgery [24].

There are potential flaws in this study, including its susceptibility to selection bias. However, we feel that this does not present a significant issue, as the allocation of method of operation was effectively random and there were no differences in patient demographics between the open and laparoscopic groups. Another potential flaw is that there was no prospective data collection on pain outcome; it is well known that retrospective data collection can be difficult due to inconsistencies in documentation. Due to the heterogeneous reporting of outcome data with regard to analgesic consumption across the literature, we have identified the used of an internationally validated Analgesic Consumption Score. This would overcome the current challenge in comparing data, and improve our ability to compare analgesic consumptions between studies in future work to evaluate these parameters. We however note that most scoring systems are subject to bias due to the subjective nature of their utilization. Furthermore, young paediatric patients incapable of using adult-type verbal scoring systems may be inconsistent in their reporting.

## Conclusion

We have shown increased postoperative pain and increased use of postoperative analgesia in children undergoing laparoscopic hernia repair compared to open repair. Caudal anaesthetic blockade decreased requirements for analgesia in recovery and the requirement for postoperative analgesia. It is therefore reasonable to suggest that caudal analgesia, where feasible, may be useful analgesic strategy in both operative modalities. Our data also suggest a small but positive impact of intraperitoneal local anaesthetic infiltration on total analgesic requirement after laparoscopic hernia repair. The use of caudal anaesthesia and local anaesthetic infiltration seems an efficacious combination in young children having laparoscopic hernia repair. A prospective evaluation of the use of these two techniques in combination in providing immediate and medium-term pain relief after laparoscopic in children may be the next step in defining optimal pain management after laparoscopic surgery in children.
